# Downregulation of L-type Ca^2+^ current in patients with atrial fibrillation results from altered channel gating and disruption of membrane microdomains

**DOI:** 10.1016/j.hrthm.2026.03.1953

**Published:** 2026-07

**Authors:** Marina Balycheva, Benedict Reilly-O’Donnell, Anita Alvarez-Laviada, Kelly Zhang, Marta Mazzola, Jose L. Sanchez-Alonso, Carla Lucarelli, Roman Y. Medvedev, Cristina E. Molina, Sophie Schobesberger, Ivan Diakonov, Peter T. Wright, Claire E. Poulet, Nadja I. Bork, Natalia A. Trayanova, Giuseppe Faggian, Prakash Punjabi, Viacheslav O. Nikolaev, Alexey V. Glukhov, Julia Gorelik

**Affiliations:** 1 National Heart and Lung Institute, Imperial College London, United Kingdom; 2 University of Verona, School of Medicine, Verona, Italy; 3 Department of Medicine, University of Wisconsin-Madison School of Medicine and Public Health, Madison, Wisconsin; 4 Department of Biomedical Engineering Johns Hopkins University, Baltimore, Maryland; 5 Humanitas Clinical and Research Center, Rozzano, Milan, Italy; 6 Institute of Experimental Cardiovascular Research, University Medical Center Hamburg-Eppendorf, Hamburg, Germany; 7 German Center for Cardiovascular Research (DZHK), partner site Hamburg/Kiel/Lübeck, Hamburg, Germany; 8 School of Life and Health Sciences, University of Roehampton, London, United Kingdom; 9 Department of Medicine, Alliance for Cardiovascular Diagnostic and Treatment Innovation, Johns Hopkins University, Baltimore, Maryland; 10 Department of Cardiothoracic Surgery, Hammersmith Hospital, National Heart and Lung Institute, Imperial College London, United Kingdom

**Keywords:** Atrial fibrillation, Cardiomyocyte, Calcium channel, Atria, Scanning ion conductance microscopy, Caveolin-3

## Abstract

**Background:**

Atrial fibrillation (AF) is associated by alterations in cardiomyocyte membrane microdomain organization that can modify the distribution and biophysical properties of L-type Ca^2+^ channels (LTCCs), contributing to downregulation of the L-type Ca^2+^ current (*I*_Ca,L_).

**Objective:**

This study aimed to examine the role of cellular microarchitecture and microdomain-specific changes of single LTCCs in *I*_Ca,L_ remodeling in chronic AF.

**Methods:**

Right atrial (RA) and left atrial (LA) biopsies from patients in sinus rhythm (SR, n = 51) and AF (n = 61) were analyzed to assess cardiomyocyte microarchitecture and microdomain-specific remodeling of single LTCCs and *I*_Ca,L_. Computational modeling estimated the contributions of different cellular components in *I*_Ca,L_ reduction.

**Results:**

In AF, *I*_Ca,L_ was reduced in RA (∼32%, *P <* .05) and LA (∼71%, *P <* .01). In RA, this was associated with preserved transverse (T)-tubular LTCCs (T-LTCCs) density and decreased extra-tubular (crest, C-LTCCs) density. In LA-AF, densities of both T- and C-LTCCs were reduced. These changes paralleled T-tubule downregulation in both atria and an LA-specific decrease in caveolin-3 expression. In AF, the open probabilities of T- and C-LTCCs in both atria were 3–5-fold higher than in SR and accompanied by 2-fold increase in protein kinase A and phosphorylated Ca^2+^/calmodulin kinase II activities. However, computational simulations showed that enhanced LTCC open probability did not compensate for reduced channel density. Caveolin-3 overexpression in LA-AF cardiomyocytes increased Ca_V_1.2 membrane expression, partially restoring *I*_Ca,L_.

**Conclusion:**

*I*_Ca,L_ downregulation in AF is driven by chamber-specific degradation of membrane structures and loss of functional LTCCs, not offset by increased channel activity. Caveolin-3 is crucial for maintaining functional LTCCs at the sarcolemma.

Atrial fibrillation (AF) is the most common cardiac arrhythmia and is associated with profound electrophysiological, functional, and structural remodeling.^1^^,^^2^ A well-established electrophysiological hallmark of atrial cardiomyocytes from both patients with AF and animal models is a marked reduction of the L-type Ca^2+^ current (*I*_Ca,L_).^3^, ^4^, ^5^, ^6^ This decrease contributes to shortening of the atrial action potential duration and effective refractory period, loss of rate-dependent action potential adaptation,^5^^,^^7^ and atrial hypocontractility.^8^ Although early studies attributed *I*_Ca,L_ downregulation to transcriptional suppression of Ca_V_1.2, the pore-forming subunit of the L-type Ca^2+^ channel (LTCC),^7^^,^^9^ subsequent investigations on human atrial tissues demonstrated preserved Ca_V_1.2 mRNA or protein expression.^5^^,^^10^ Instead, reduced *I*_Ca,L_ has been linked to impaired basal LTCC phosphorylation because of increased phosphatase activity.^5^ However, this interpretation is difficult to reconcile with single-channel recordings showing increased open probability of individual LTCCs in AF.^9^

The coexistence of elevated single-channel activity with diminished whole-cell *I*_Ca,L_ suggests an alternative mechanism: a reduction in the number of functional LTCCs available at the cardiomyocyte surface. Direct testing of this hypothesis has been limited by the lack of methodologies capable of resolving LTCC activity within defined sarcolemmal microdomains in intact human cardiomyocytes. To address this gap, the present study employed a super-resolution scanning patch-clamp technique^11^ that enables nanoscale mapping of membrane architecture together with functional interrogation of individual LTCCs in situ. Using this approach, we previously identified 2 spatially distinct and approximately equally abundant LTCC subpopulations in human atrial cardiomyocytes: channels localized within transverse (T)-tubules (T-LTCCs) and channels residing on domed crest regions of the sarcolemma (C-LTCCs), the latter associated with caveolar membrane structures.^12^^,^^13^ Despite their potential importance, how these LTCC subpopulations remodel in human AF has not been systematically examined.

An additional limitation of prior work is the lack of regional resolution. Most studies of *I*_Ca,L_ remodeling have focused exclusively on right atrial (RA) appendage biopsies^5^^,^^6^^,^^8^, ^9^, ^10^^,^^14^^,^^15^ or pooled samples from both atria,^4^^,^^7^ precluding assessment of atrium-specific mechanisms. Consequently, it remains unclear whether *I*_Ca,L_ remodeling is comparable between the RA and left atria (LA) and whether the underlying molecular- and microdomain-level mechanisms are shared. Although AF is a global atrial disease involving widespread electrical, structural, and Ca^2+^-handling remodeling, substantial evidence indicates pronounced regional heterogeneity. Differences in *I*_Ca,L_ downregulation across atrial regions may differentially contribute to action potential shortening, promoting triggered activity through Ca^2+^-dependent delayed after depolarizations and facilitating re-entry by shortening the wavelength for propagation. Although arrhythmogenic triggers often localize to specific structures such as the pulmonary veins, re-entrant circuits, and conduction abnormalities are distributed throughout both atria. Moreover, *I*_Ca,L_ downregulation plays a central role in atrial hypo-contractility, which underlies impaired atrial hemodynamics and thrombus formation, which is preferably linked to atrial appendages. Together, these observations underscore the importance of understanding region-specific remodeling of *I*_Ca,L_ in human AF.

In the present study, we integrated high-resolution experimental measurements with computational modeling to demonstrate that *I*_Ca,L_ downregulation in AF: (1) occurs in both atria, with a more pronounced reduction in the LA; (2) is driven by chamber-specific, stress-induced degradation of membrane structures—including T-tubules and caveolin-3 enriched microdomains—resulting in a reduced number of functional LTCCs; and (3) is not compensated by the concomitant increase in channel open probability. Specifically, we proposed that the reduction in C-LTCCs density is likely linked to the loss of caveolar structures in both atria, whereas the LA-specific decrease in T-LTCC density correlates with decreased expression of the caveolar scaffolding protein caveolin-3, a change not observed in the RA. Finally, we show that overexpression of caveolin-3 protein in LA-AF cardiomyocytes increases Ca_V_1.2 surface expression and partially restores *I*_Ca,L_, underscoring the role of caveolin-3 in maintaining functional LTCCs at the sarcolemma.

## Methods

Detailed methods are available in the Supplementary Methods.

### Ethical approval

All the institutional review board (IRB) protocols were approved by the corresponding IRBs. Human atrial samples were obtained from patients undergoing coronary artery bypass surgery and/or mitral valve repair/replacement with or without concomitant AF ablation at Hammersmith Hospital, United Kingdom (Ethical approval number 12/WA/0196, REC Wales) and University of Verona, Italy (Prof.847CESC-Prot.13371 AOUI), and have therefore been performed in accordance with the ethical standards laid down in the 1964 Declaration of Helsinki and its later amendments. All persons gave their informed consent prior to their inclusion in the study. Normal human hearts that went unused for organ transplant were obtained from the University of Wisconsin Organ Procurement Organization, Madison, Wisconsin, as approved by the University of Wisconsin IRB.

### Patients

112 patients with preserved left ventricular function were studied. There were 51 patients with sinus rhythm (SR) and 61 male and female patients with AF (Table 1). All AF and 43 SR tissue samples were harvested from patients undergoing open heart surgery; an additional 8 SR tissue samples were collected from unused donor hearts. Samples of RA and/or LA appendages were collected and used for myocyte isolation or were flash-frozen in liquid nitrogen for biochemical studies.

**Table 1:** Clinical characteristics of patients and pathology of atrial tissue used in functional and structural studies

Clinical details	SR	AF	*P*-value
N	51	61	-
Sex, M/F	34/17	38/23	.694
Age, years	58–81	61–83	
CABG, n (%)	17 (33%)	12 (20%)	.100
MVR, n (%)	26 (50%)	49 (80%)	.001
Discarded donor heart	8 (17%)	0 (%)	.001
EF, %	> 60	>55	
LA diameter, mm	44.9 ± 6.1	53.2 ± 9.3	.001
RA short-axis diameter, mm	46.1 ± 4.6	51.2 ± 6.2	.002
Calcium channel blockers, %	15 (29%)	9 (15%)	.069
Beta-blockers, %	25 (49%)	53 (87%)	<.001

AF = atrial fibrillation; Beta-blockers = beta-adrenergic receptors blockers; CABG = coronary artery bypass grafting; EF = ejection fraction; LA = left atrium; MVR = mitral valve repair/replacement surgery; RA = right atrium; SR = sinus rhythm.

### Scanning ion conductance microscopy and super-resolution scanning patch-clamp

For single LTCC measurements, surface topography was first visualized by scanning ion conductance microscopy (SICM), which uses a glass nano-pipette as a sensitive probe.^16^ SICM is a non-contact scanning probe microscopy technique based on the principle that the flow of ions through the tip of a nano-pipette decreases when the nano-pipette approaches the surface of the sample.^17^ After generating a topographical image of the cell surface, the tip diameter of the pipette was widened by clipping to increase the area of attachment. The pipette was then lowered to a specific location until it touched the membrane, and a high resistance seal was established. Recordings were then performed in a cell-attached mode as previously described.^12^

### Computational modeling of changes associated with AF

To determine the influence of different biophysical and cell ultrastructure parameters on the outcome of global calcium entry in AF myocytes, we employed computer simulations. The Grandi et al^18^ human atrial model, which contains 2 discrete domains for *I*_Ca_, in the junctions (T-tubules) and at the sarcolemma (crest), was implemented to represent cell behavior in SR, for both LA and RA myocytes. T-tubule density and LTCC occurrence were incorporated as new microdomain parameters, which fractionally modified the LTCC current, and sodium and potassium leak currents. Additionally, the LTCC gating parameters d_ss_ and τ_f_ were modified in the model to account for observed differences between RA and LA currents in AF. Finally, modifications were implemented to increase the channel opening gate (d). This was introduced to account for the reduced mean close time, reported in our experiments. Detailed description of computational modeling is available in the online Supplementary Materials.

### Statistical analysis

Continuous variables were summarized as mean ± standard error of the mean for the given number of experiments, where N denoted the number of patient samples and n refers to the number of measurements; categorical variables were shown as frequency and percentage. Statistical analysis was carried out using an unpaired Student *t* test or Mann-Whitney after test for normality (Shapiro-Wilks test). Categorical variables were compared with Fisher’s exact test. A value of *P <* .05 was considered as significant. Where more than 2 unrelated conditions were evaluated, a 1-way analysis of variance (ANOVA) with a Bonferroni’s post hoc test was applied.

## Results

### Study population

Patient characteristics are summarized in Table 1. Age range and sex distribution were similar between groups. All patients had a preserved left ventricular ejection fraction, which was an inclusion criterion. The type of cardiac surgery and underlying cardiac pathology differed between groups. Mitral valve surgery was the most common intervention in the AF group (80%), whereas coronary artery bypass grafting was equally represented in both groups (*P* = .1). Healthy donor hearts discarded from the organ donation program constituted a small but significant portion of the SR group. Atrial size was significantly larger in the AF group both for the RA (*P* = .002) and LA (*P* = .001). Beta-blocker use was significantly higher in the AF group (*P <* .001), whereas calcium channel blockers were more frequently used in patients undergoing coronary artery bypass grafting, although this difference did not reach statistical significance (*P* = .069).

### Decrease in I_Ca,L_ in AF is associated with reduced sarcolemmal LTCC occurrence

**Figure 1:**
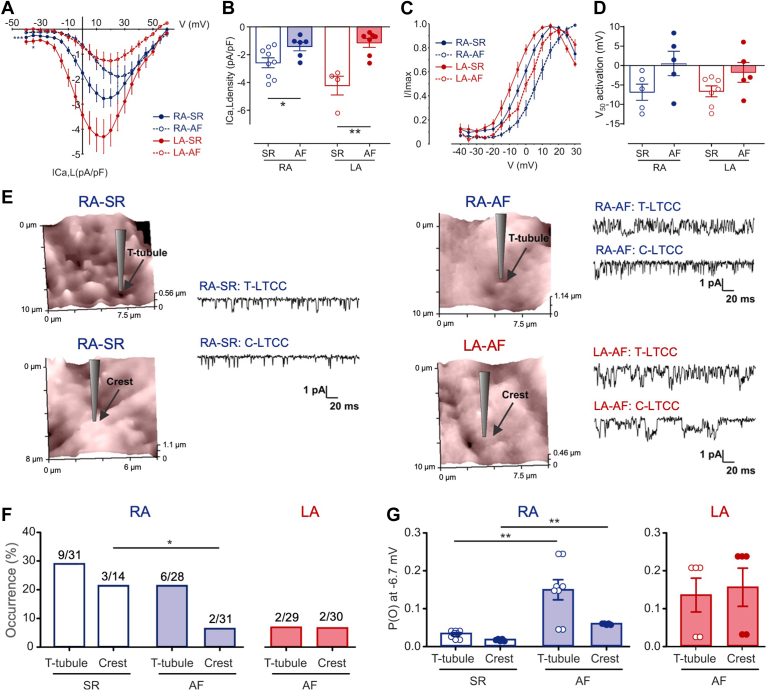
Whole-cell and single-channel *I*_Ca,L_ current measured in RA and LA myocytes from patients with SR and AF. Current–voltage relationship of the whole-cell *I*_Ca,L_ (**A**), steady-state curves of voltage-dependent *I*_Ca,L_ activation (**B**), mean basal current density measured at +10 mV (**C**), and mean V50 activation voltages (**D**) measured in cardiomyocytes isolated from RA-SR (N/n = 4/9), RA-AF (N/n = 2/6), LA-SR (N/n = 4/4) and LA-AF (N/n = 4/7) samples. ∗*P* < .05, ∗∗*P* < .01 for SR vs AF by 1-way ANOVA with Tukey’s post-hoc test. **E:** Representative SICM surface topography scans are shown for RA-SR, RA-AF, and LA-AF myocytes; T-tubule and crest regions from which channel activity were recorded (T-LTCC and C-LTCC, respectively), are indicated. On the right, representative single channel traces recorded at −6.7 mV are shown to illustrate an enhanced LTCC activity (ie, more often and longer channel openings) in AF myocytes. **F:** A single-channel LTCC current distribution (% of occurrence) in human RA and in LA myocytes obtained from patients with SR and AF. ∗*P <* .05 by Fisher exact test. **G:** Open probability P_O_ measured at −6.7 mV in RA and LA myocytes in SR and AF (RA-SR: T-LTCC = 12 and C-LTCC = 5 channels; RA-AF: T-LTCC = 8 and C-LTCC = 5 channels; LA-AF: T-LTCC = 5 and C-LTCC = 5 channels). ∗*P <* .05 and ∗∗∗*P* < .001 by Fisher’s exact test. AF = atrial fibrillation; ANOVA = analysis of variance; LA = left atrium; LTCC = L-type Ca^2+^ channels; PO = open probability of single LTCC; RA = right atrium; SR = sinus rhythm.

Peak *I*_Ca,L_ amplitude was significantly reduced in both RA-AF and LA-AF myocytes compared with corresponding SR cells, with a more pronounced reduction in the LA (∼71%) than in the RA (∼32%; Figure 1A and 1B). No statistically significant difference in the midpoint of *I*_Ca,L_ activation was found between the SR and AF groups (Figure 1C and 1D). To determine whether whole-cell *I*_Ca,L_ downregulation reflected changes at the single-channel level, we applied SICM combined with super-resolution patch-clamp. As previously shown in rat and human atrial myocytes,^12^ functional LTCCs were detected both within T-tubules and on the sarcolemmal crest in SR and AF samples from both atria (Figure 1E). In RA-SR, LTCCs were evenly distributed between T-LTCC and C-LTCC locations. In RA-AF cells, T-LTCC occurrence was preserved, whereas C-LTCC occurrence was significantly reduced compared with RA-SR (Figure 1F, left). In LA-AF myocytes, both T- and C-LTCCs were markedly less frequently detected than in RA-SR cells (6.9% and 3.3%, respectively; Figure 1F, right), indicating a substantial reduction in the number of functional LTCCs available at the membrane. LA-SR data was not available for all experiments. This is because of limited tissue availability and the proximity of laboratories to the clinical sites.

### Augmented LTCC activity in AF

Despite reduced channel occurrence, LTCC activity was increased in AF. In RA-AF myocytes, both T- and C-LTCCs exhibited significantly higher open probability (P_O_), with a 4.5-fold increase for T-LTCC (from 0.033 ± 0.002 in SR to 0.149 ± 0.027 in AF, *P <* .01) and a 3.5-fold increase for C-LTCC (from 0.017 ± 0.001 in SR to 0.059 ± 0.001 in AF, *P <* .01; Figure 1G, left, and Table S1). This increase in P_O_ resulted primarily from shortened mean closed times, without significant changes in mean open times (Table S1). In both SR and AF, multichannel openings were observed. Open-time histograms revealed that in SR, most T-LTCC openings were brief (<1 ms, mode 1), whereas prolonged openings (>1 ms, mode 2) were rare (Figure S1A). In AF, the probability of long openings increased markedly (92%, Figure S1B). Closed-time histograms demonstrate a rescued probability of closed states >5 ms in AF, likely contributing to increased channel availability. In contrast, C-LTCCs in AF additionally exhibited an increased frequency of high-amplitude sub-conductance openings. The probability of C-LTCC openings in modes 1 and 2 increased by 21% and 57% in AF, respectively (Figure S2).

In LA-AF, P_O_ of the remaining T- and C-LTCCs was also significantly higher than in RA-SR cells (Figure 1G, right). Similar to RA-AF, T- and C-LTCCs in LA-AF displayed comparable probabilities of modes 1 and 2 openings, whereas the probability of the closed state was approximately 5-fold higher for T-LTCC than for C-LTCC (Figure S3). Single-channel recordings demonstrated predominantly active channels in AF, compared with both active and silent channels in SR (Figure 1E). Importantly, single-channel conductance and amplitude were unchanged (Table S1), indicating preserved biophysical properties.

### Upregulation of PKA and CaMKII activity in AF

**Figure 2:**
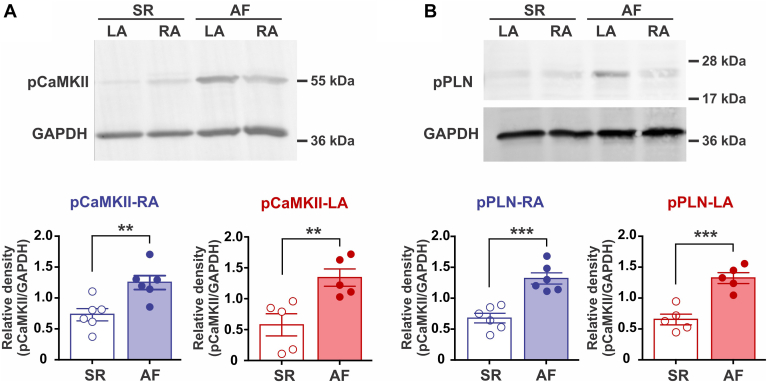
Upregulation of PKA and CaMKII activity in AF. Western blot of human atrial tissue lysates probed for (**A**) auto-phosphorylated at the Thr^286/287^ site CaMKII, and (**B**) PKA-dependent phosphorylated site Ser^16^ of phospholamban. Statistical significance was assessed by unpaired Student’s *t* test, *P <* .001, n = 5–6 per diagnosis, 3 technical replicates per blot. AF = atrial fibrillation; CaMKII = Ca^2+^-calmodulin kinase II; LA = left atrium; LTCC = L-type Ca^2+^ channels; PKA = protein kinase A; RA = right atrium; SR = sinus rhythm.

The increased LTCC P_O_ in AF is consistent with enhanced channel phosphorylation.^19^ Both Ca^2+^-calmodulin kinase II (CaMKII) and protein kinase A (PKA) are known regulators of LTCC activity. To assess their involvement, we quantified expression of auto-phosphorylated CaMKII (pCaMKII, Thr^286/287^) and PKA-dependent phospholamban phosphorylation at Ser.^16^ Both pCaMKII and PLB-Ser^16^ were significantly upregulated in RA and LA AF samples (Figure 2), supporting a phosphorylation-dependent mechanism underlying increased LTCC activity in AF. These findings are consistent with our previous observations in ventricular myocytes from patients with cardiomyopathies.^20^

### Beta-adrenergic modulation of LTCC current

**Figure 3:**
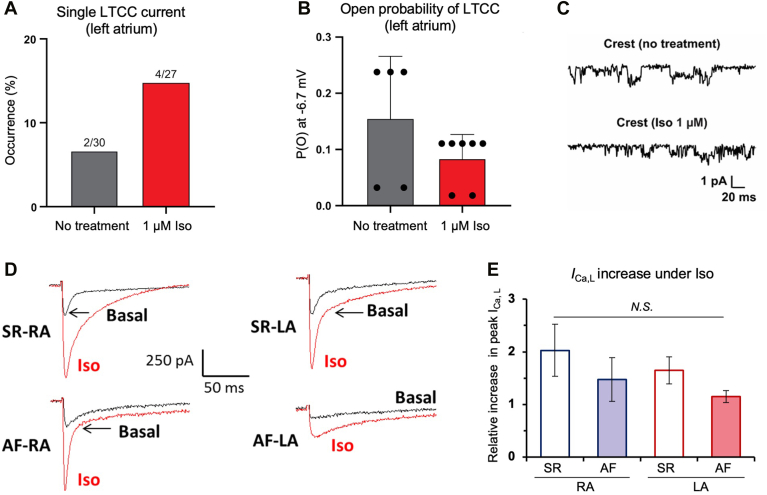
Effect of isoproterenol (Iso, 1 mM) stimulation on single C-LTCCs in LA-AF myocytes and on the whole-cell *I*_*Ca,L*_ in RA and LA myocytes from patients with SR and AF. **A:** Single C-LTCC current distribution (% of occurrence) recorded in the crest microdomain. **B**: Open probability (P_O_) measured at −6.7 mV without (no treatment, n = 5 channels) and with 1 μM Iso (n = 7 channels), *NS*. **C:** Representative single channel traces at −6.7 mV showing LTCC activity recorded in the crest without and with Iso. **D:** Representative whole-cell peak *I*_Ca,L_ traces in the presence and absence of Iso. **E:** Relative increase in the peak *I*_Ca,L_ current density in the presence of Iso compared with basal current from RA-SR (n = 12), RA-AF (n = 8), LA-SR (n = 13) and LA-AF (n = 5) myocytes. Significance was assessed by 1-way ANOVA with Tukey’s post hoc test. AF = atrial fibrillation; ANOVA = analysis of variance; LA = left atrium; LTCC = L-type Ca^2+^ channels; RA = right atrium; SR = sinus rhythm.

Consistent with previous reports, sympathetic regulation of atrial *I*_Ca,L_ was preserved in AF.^5^^,^^21^ This may indicate a further increase in LTCC P_O_ or recruitment of additional “silent” channels during sympathetic stimulation. In our experiments, application of isoproterenol (1 μM in the bath and 1.5 μM in the patch pipette) significantly increased occurrence of C-LTCC in LA-AF myocytes from 6.7% (2 single LTCC activities of 30 patches) to 14.8% (4 single LTCC activities of 27 patches) (Figure 3A), without changing P_O_ (0.15 ± 0.05 vs 0.084 ± 0.02 in untreated vs. isoproterenol-treated cells, *P* = .238, Figure 3B and 3C).Whole-cell recordings revealed increased *I*_Ca,L_ amplitude and current density in isoproterenol-stimulated myocytes across all groups (Figure 3D and 3E). No statistically significant difference was found between cell groups stimulated with isoproterenol. Together, these results argue against the presence of a large reserve of non-functional “silent” channels that could be recruited under sympathetic stimulation and instead suggest impaired LTCC trafficking to the membrane as a major contributor to *I*_Ca,L_ downregulation in AF.

### Degradation of structural microdomains in AF

Unlike ventricular myocytes, where most of the LTCCs are associated with T-tubules,^11^^,^^20^^,^^22^ atrial LTCCs are evenly distributed between T-tubules (T-LTCC) and extra-tubular regions (C-LTCC).^12^ Extra-tubular channels are believed to be associated with caveolae structures and can contribute to up to 50% of the whole-cell *I*_Ca,L_ in atrial myocytes,^12^ whereas in ventricular myocytes, C-LTCCs are responsible for about 15% to the whole-cell *I*_Ca,L_.^13^ Profound AF-induced remodeling of membrane microdomains^23^^,^^24^ could therefore differently affect these LTCC populations, with a varying impact on *I*_Ca,L_. To assess this further, we assessed T-tubule organization and expression of caveolar scaffolding protein, caveolin-3, and then implemented a computational modeling to estimate the contribution of different cellular components into *I*_Ca,L_ downregulation in both atria.

**Figure 4:**
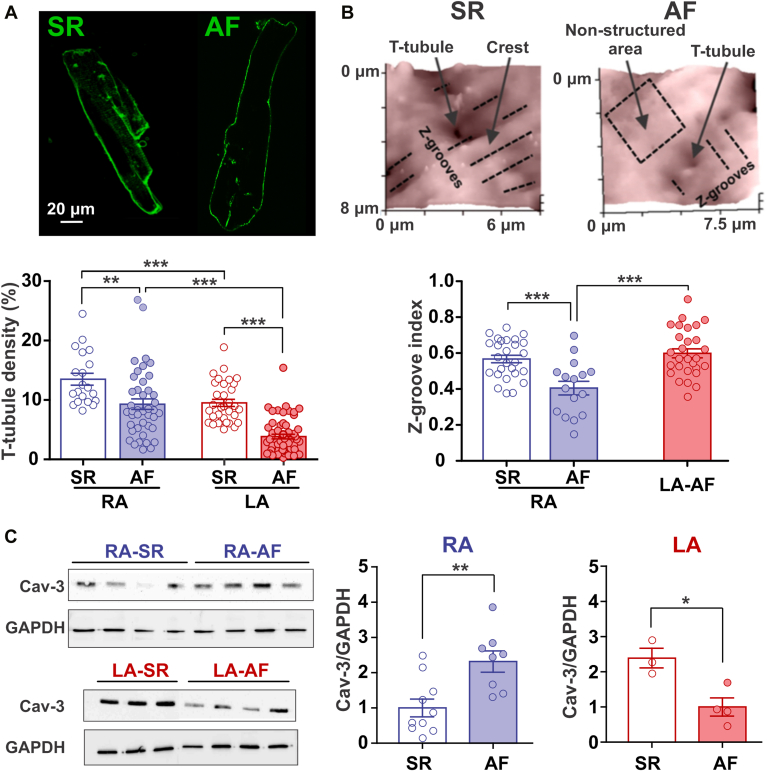
Transverse T-tubules, surface topography and caveolae integrity in human RA and LA myocytes from patients with SR and AF. T-tubules network (**A**) and cardiomyocyte surface topography scans (**B**) of atrial myocytes from the patients with SR and AF. T-tubules, crests, and non-structured areas are indicated by *arrows*. Below, average data for T-tubule density (as % of cell volume) and Z-groove index (calculated as a ratio of z-grooves per 10 μm x 10 μm scan) are shown. ∗∗*P <* .01, ∗∗∗*P* < .001 for SR vs AF groups. *P*-values were determined by 2-way ANOVA with Bonferroni correction for T-tubule density and by Student’s *t* test for Z-groove index. **C:** Representative caveolin-3 (Cav-3) immunoblots from SR and AF atrial tissue homogenates and GAPDH-normalized Cav-3 levels from RA (n = 10/9 SR/AF) and LA (n = 3/4 SR/AF) tissues, 3 technical replicates per blot. ∗*P <* .05, ∗∗*P* < .01, and ∗∗∗*P* < .001, respectively, for AF vs SR by Student *t* test. AF = atrial fibrillation; ANOVA = analysis of variance; LA = left atrium; RA = right atrium; SR = sinus rhythm.

We found that most SR atrial myocytes exhibited irregular T-tubule organization (Figure 4A). AF caused a significant reduction in T-tubule density in both atria, with more severe degradation in LA-AF cells (∼60%) than in RA-AF cells (∼31%), consistent with the greater loss of T-LTCC LA-AF (Figure 1F, right). These changes were accompanied by disrupted surface topography (characterized by the Z-groove index calculated as a ratio of Z-groove length to a total extrapolated Z-groove length, Figure 4B) and a loss of T-tubule openings (data not shown). Notably, caveolin-3, which plays an important role in both caveolae formation and T-tubule integrity,^25^, ^26^, ^27^ was selectively downregulated in LA-AF but upregulated in RA-AF (Figure 4C), indicating chamber-specific remodeling.

### Computational modeling of AF-induced LTCC remodeling

**Figure 5:**
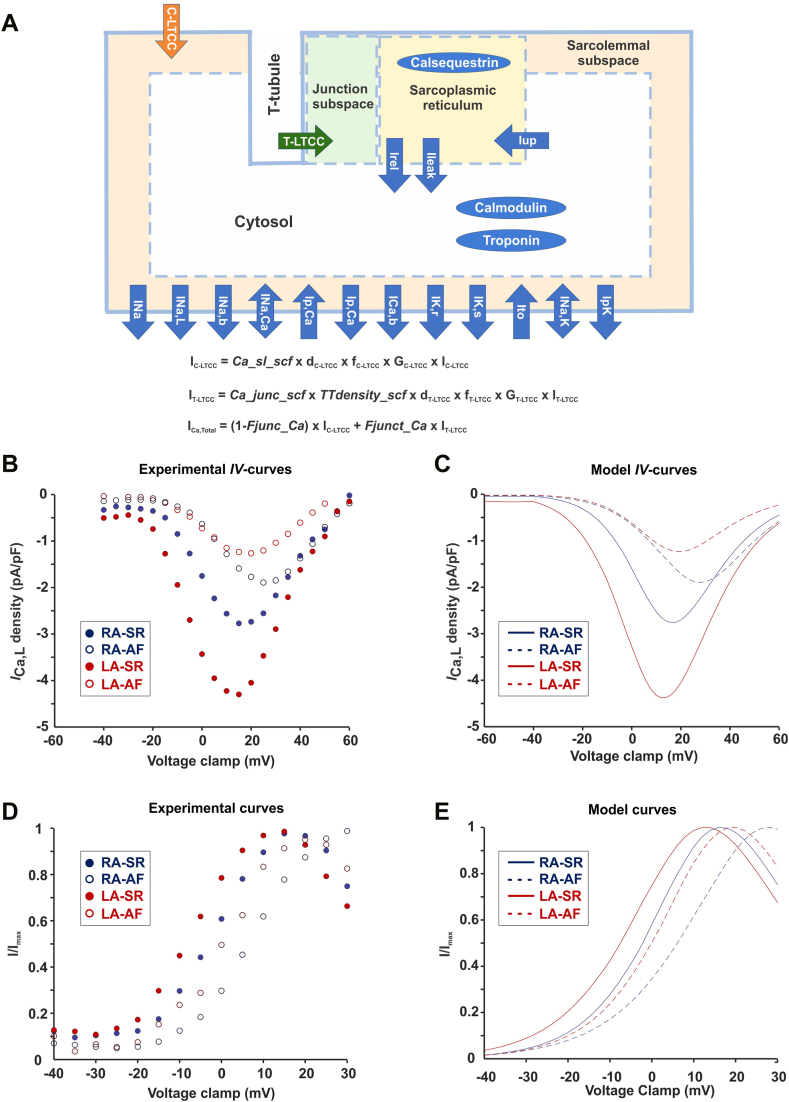
Computational modeling of experimentally observed *I*_Ca,L_ downregulation in right (RA) and left (LA) atria in AF. **(A)** The human atrial AP and Ca^2+^ model includes subspaces for the cytosol, sarcoplasmic reticulum, tubule junction, and sarcolemma. The gating equations (d_C/T-LTCC_, f_C/T-LTCC_) and channel conductances (G_C/T-LTCC_) were modified to account for microdomain differences. Additional parameters that modify the current because of changes in T-tubule density (*TTdensity_scf*), microdomain occurrence (*Ca_sl_scf*, *Ca_junc_scf*), and ratio of T-tubule to crest channels (*Fjunc_Ca)* are described in the main text. The baseline current equations (Ī_C/T-LTCC_) were unmodified from Grandi et al^18^**B through E:** Experimental vs model I–V curves (**B and C**) and steady-state curves of voltage-dependent channel activation (**D and E**) in RA and LA myocytes from patients with SR and AF. Points are experimental data; lines are model results. AF = atrial fibrillation; LA = left atrium; LTCC = L-type Ca^2+^ channels; RA = right atrium; SR = sinus rhythm.

**Figure 6:**
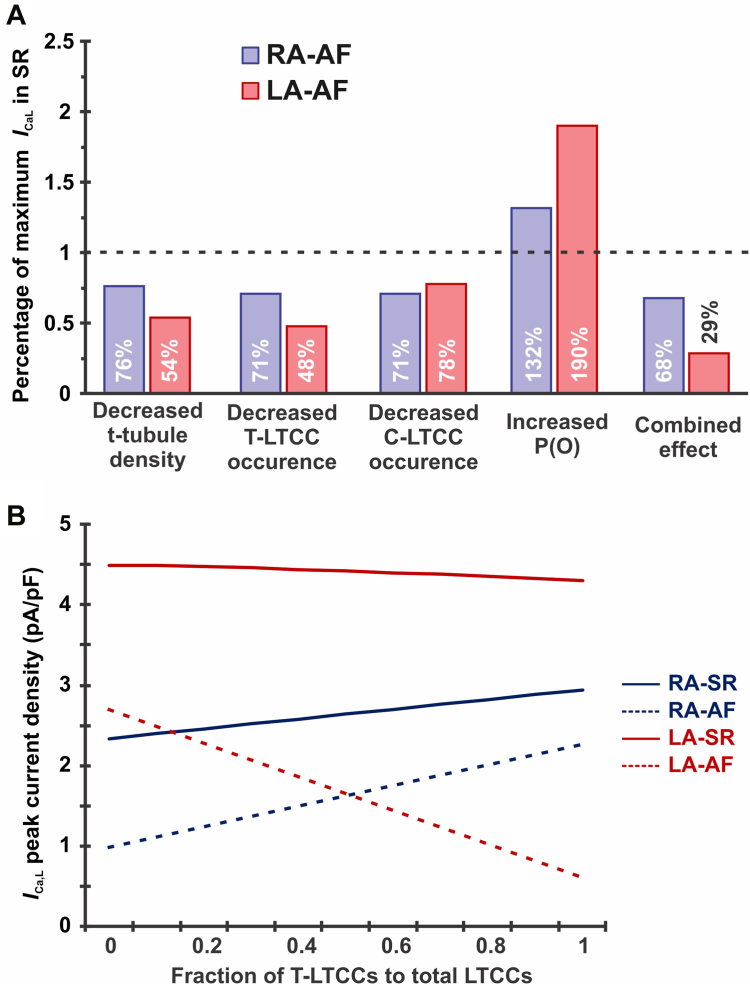
Relative impact of individual ultrastructural and biophysical changes on *I*_Ca,L_ remodeling in right (RA) and left (LA) atrial cardiomyocytes during AF. Whole-cell *I*_Ca,L_ computational modeling incorporating structural and biophysical channel modulation in AF. **A:** Contribution of individual changes and their cumulative effect on the maximum current amplitude in AF at membrane potential V_m_ = 0 is shown. **B:** Contribution of T-LTCC vs C-LTCC to the *I*_Ca,L_ peak current in SR and AF right and left atrial myocytes. AF = atrial fibrillation; LTCC = L-type Ca^2+^ channels; SR = sinus rhythm.

To integrate these findings, we used a modified the human atrial ionic model by Grandi et al^18^ to reproduce experimentally measured LTCC behavior for RA and LA myocytes in SR, assuming similar channel content for T-LTCCs and C-LTCCs^28^ (Figure 5A). Using parameters reflecting our experimental findings in AF cardiomyocytes, we reproduced both a reduction in peak whole-cell *I*_Ca,L_ and a positive shift of the maximum current in both RA-AF and LA-AF (Figure 5B and 5C). The calculated *I-V* curves for RA and LA myocytes are shown in Figure 5C. Figure 6A presents the breakdown of the contribution of the individual AF-induced changes to *I*_Ca_ in AF, including decrease on T-tubule density, decrease of T- and C-LTCC occurrence, and an increase in their P_O_. Our simulations demonstrate that downregulation of *I*_Ca,L_ in both atria AF is driven primarily by chamber-specific loss of membrane microdomains, including caveolae and T-tubules, and the associated decrease in the occurrence of functional LTCCs, which is not compensated by increased channel activity (Figure 6A). Although T- and C-LTCCs contributed equally to *I*_Ca,L_ in SR, AF remodeling shifted this balance: T-LTCC loss dominated *I*_Ca,L_ reduction in LA, whereas C-LTCC loss was more influential in RA (Figure 6B).

### Overexpression of caveolin-3 partially restores I_Ca,L_ in AF myocytes

**Figure 7:**
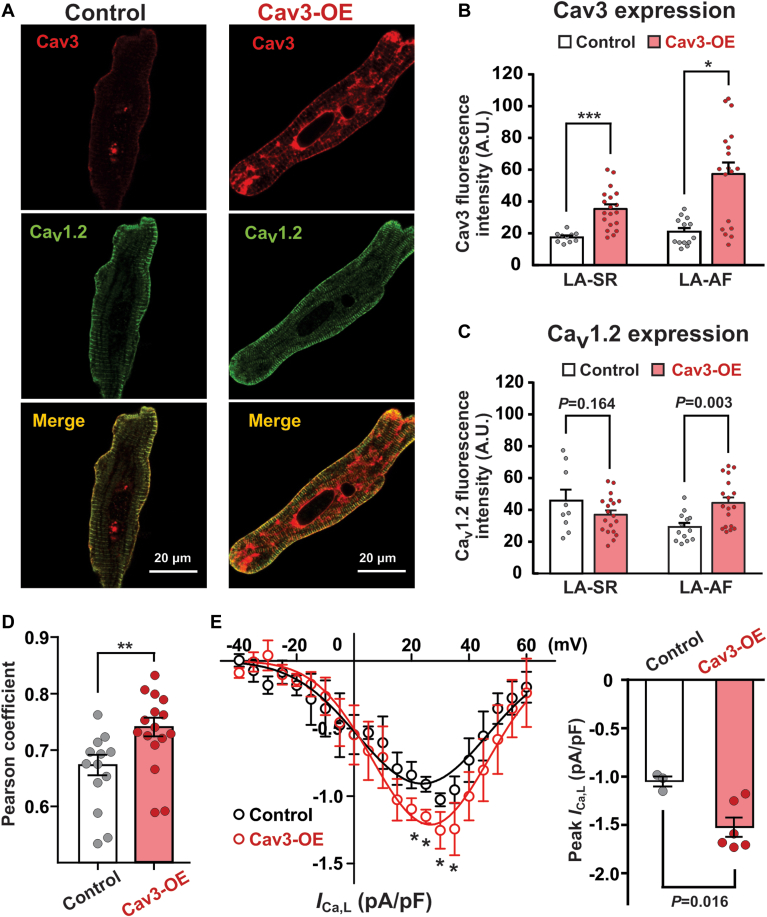
Overexpression of caveolin-3 upregulates membrane level of Ca_v_1.2 and partially restores *I*_Ca,L_ in LA-AF myocytes. **A:** Immunofluorescent staining of caveolin-3 (Cav3, *red*) and Ca_V_1.2 (*green*) in control (LA-AF, 48-hour culture, but not transfected) and caveolin-3 overexpression (LA-AF with Cav3-OE) left atrial human myocytes from patients with AF. **B and C:** Relative expression of caveolin-3 (**B**) and Ca_V_1.2 (**C**) is shown for LA-AF (25 cells/3 patients and 43 cells/3 patients for 48-hour cultured control and Cav3-OE groups, respectively) and LA-SR (10 cells/1 patient and 19 cells/1 patient for 48-hour cultured control and Cav3-OE groups, respectively) groups. Statistics by Mann-Whitney after Shapiro-Wilk normality test. **D:** Individual Pearson coefficients are shown for LA-AF cardiomyocytes transfected with caveolin-3 (Cav3-OE, n = 10 cells from n = 3 samples) and non-transfected, but cultured (control, n = 10 cells from n = 2 samples). ∗*P <* .05 by unpaired Student’s *t* test. **E:** Current–voltage relationship of *I*_Ca,L_ and average peak *I*_Ca,L_ current in cultured, but not transfected (control, n = 6 cells) and transfected with Cav3 (Cav3-OE, n = 3 cells) LA-AF myocytes. ∗*P* < .05 by unpaired Student’s *t* test. AF = atrial fibrillation; LA = left atrium; LTCC = L-type Ca^2+^ channels; RA = right atrium; SR = sinus rhythm.

Because caveolin-3 was selectively downregulated in LA-AF, we tested whether its overexpression could restore *I*_Ca,L_. Cardiomyocytes isolated from LA-SR and LA-AF samples, were infected with an adenovirus expressing caveolin-3. 40–56 hours post-infection, the whole-cell *I*_Ca,L_ was measured in cells transfected with caveolin-3 and compared with cells transfected with empty virus. As expected, caveolin-3 expression level was significantly upregulated in cells transfected with caveolin-3 virus compared with cells transfected with empty virus in all groups of cells (Figure 7A and 7B). This was accompanied by significant (*P <* .05) increase in Ca_V_1.2 level found specifically in LA-AF but not in LA-SR cells (Figure 7C). Caveolin-3 overexpression also increased co-localization between caveolin-3 and Ca_V_1.2 in LA-AF cells (0.73 ± 0.06 vs 0.66 ± 0.09 in caveolin-3 transfected [n/N = 10/3] vs non-transfected [n/N = 10/2] cells, *P <* .01; Figure 7D). Importantly, caveolin-3 overexpression significantly increased peak *I*_Ca,L_ by 60% (*P <* .05), partially restoring current density toward LA-SR levels (Figure 7E). These data provide the first proof-of-concept evidence that disruption of membrane microdomains—and their scaffolding proteins—plays a central role in LTCC remodeling and *I*_Ca,L_ downregulation in human AF, identifying caveolin-3-dependent channel trafficking as a potential therapeutic target.

## Discussion

Downregulation of *I*_Ca,L_ is a hallmark of electrical remodeling in patients with AF, contributing to shortening of the atrial effective refractory period and loss of physiological rate adaptation,^5^^,^^7^ thereby promoting arrhythmia maintenance and resistance to treatment. Despite the well-recognized importance of *I*_Ca,L_ downregulation in AF, the underlying cellular and molecular mechanisms have remained incompletely understood. This gap has been complicated, in part, by the apparent contradiction between unchanged mRNA and protein expression of LTCC subunits (including Ca_V_1.2 and Ca_V_b_2a_)^5^ and significantly increased single-channel activity reported in patients with AF.^9^ Although these observations suggest a reduction in the number of available functional channels in the cardiomyocyte sarcolemma, this hypothesis could not be directly tested until recently because of the lack of appropriate techniques to probe LTCC compartmentalization and functionality at the single-channel level.

Here, we employed super-resolution scanning patch-clamp to visualize sarcolemmal structures in live cardiomyocytes at nanoscale resolution and to probe functional LTCCs within distinct membrane microdomains. We found that in patients with chronic AF, *I*_Ca,L_ downregulation occurs in both atria, with a more pronounced reduction in the LA, and is associated with degradation of membrane structures and a concomitant decrease in the occurrence of functional LTCCs, without full compensation by enhanced channel activity. Specifically, our cumulative results suggest that the decrease in the density of extra-tubular LTCCs (C-LTCCs), observed in both atria, may be driven by downregulation of caveolae structures, similar to what has been reported for C-LTCC in ventricular myocytes^13^ and sinoatrial node cells.^29^ Furthermore, a decrease in T-LTCC density, which appears to be present in LA-AF cells, could be associated with atrium-specific downregulation of caveolin-3 expression, a factor known to influence LTCC density within T-tubules,^27^ and was not observed in RA-AF samples. Because single-channel recordings from LA-AF cells were not available, these atrium-specific interpretations should be considered with appropriate caution.

The critical role of caveolin-3 in maintaining functional LTCCs on the cardiomyocyte sarcolemma was further supported by experiments in cultured cardiomyocytes. Overexpression of caveolin-3 in LA-AF myocytes significantly increased Ca_V_1.2 expression, enhanced co-localization between caveolin-3 and Ca_V_1.2, and partially restored whole-cell *I*_Ca,L_ (Figure 7). These findings are consistent with previous studies demonstrating a key role of caveolin-3 in LTCC trafficking^30^ and in supporting the T-tubular component of *I*_Ca,L_.^27^ Downregulation of caveolin-3 in atrial tissues has been reported in several pathological conditions, including AF,^14^ heart failure,^29^ and chronic hypertension,^24^ and may contribute to disruption of both T-tubules^27^ and caveolae.^26^ Caveolae may also be disrupted independently of caveolin-3 expression by chronically elevated membrane tension in response to atrial volume and pressure overload,^24^^,^^31^, ^32^, ^33^ a common feature of AF.^2^

In addition to LTCCs, caveolin-3 organize multiple signaling molecules, including components of the β_2_-adrenergic receptor signaling cascade such as adenylyl cyclase 5/6, PKA, and protein phosphatase 2A (PP2A).^34^ Caveolin-3 has been implicated in regulating β_2_-adrenergic receptor-associated cyclic adenosine monophosphate (cAMP) signaling by confining adenylyl cyclases and phosphodiesterases within caveolae nanodomains, thereby limiting cAMP diffusion.^31^^,^^35^ We and others have shown that downregulation of caveolin-3 in failing ventricular myocytes,^36^ expression of dominant negative caveolin-3 mutants,^37^ or acute disruption of caveolae via stretch or cholesterol depletion^31^^,^^38^ converts localized cAMP signaling into a global cytosolic signal, altering downstream phosphorylation targets.

In human AF, downregulation of phosphodiesterase 4 elevates cAMP levels, increasing Ca^2+^ spark frequency and arrhythmias susceptibility in atrial tissues.^39^ Abnormal cAMP signaling may stimulate CaMKII and PKA activity, as observed in both RA and LA biopsies from patients with AF (Figure 3), and contribute to increased availability and open probability of both T- and C-LTCCs (Figure 1G). In failing human ventricular myocytes, we previously linked the increased T-LTCC P_O_ to elevated PKA activity, whereas increased C-LTCC activity was associated with CaMKII activation.^20^ Microdomain-specific increase in PKA activity within T-tubules was also been shown to locally enhance of T-LTCC, but not C-LTCC, activity in failing right ventricular rat myocytes.^40^ Conversely, increased CaMKII activity preferentially upregulates C-LTCC,^22^ potentially through caveolae-associated nanodomains.^41^

The increase in LTCC P_O_ in AF was associated with shortened mean closed time, without changes in mean open time. Channels exhibited increased occupancy of both short (mode 1) and long (mode 2) open gating states. Elevated mode 2 gating has been linked to enhanced PKA-dependent LTCC phosphorylation.^42^ Alternatively, increased LTCC activity in AF has been attributed to reduced dephosphorylation resulting from decreased phosphatase PP2A/PP1 activity.^9^ In contrast, other studies have reported increased phosphatase activity in AF.^5^ Notably, pharmacological inhibition of PP2A/PP1 with okadaic acid prolongs mean open time without affecting closed time,^43^ which differs from the gating behavior observed in the present study (Table S1). These discrepancies highlight the need for future studies examining microdomain-specific regulation of LTCC phosphorylation machinery in the RA and LA separately.

### Study limitations

Limitations of this study primarily relate to sample type and availability. The cohort included patients with significant mitral valve and coronary artery disease (Table 1), and underlying cardiac pathology may contribute to variability in *I*_Ca,L_ remodeling during AF and between atria.^2^ The molecular basis of *I*_Ca,L_ downregulation in AF is complex and likely influenced by comorbid conditions; therefore, the observed changes cannot be attributed exclusively to AF. Atrial tissue was limited to RA and LA appendages, the only atrial regions obtainable from living patients. Although appendages recapitulate key features of atrial remodeling, regional heterogeneity in *I*_Ca,L_ remodeling across the atria could not be fully assessed. In addition, atrium-matched SR and AF samples were not available for all experimental modalities, including single-channel recordings, limiting direct comparisons in some analyses. The present study was performed using mixed male and female SR and AF samples. Although sex-specific analyses were not feasible given sample availability, emerging evidence suggests that perturbations in calcium homeostasis in AF, including *I*_Ca,L_ downregulation, may be sex-dependent.^44^ Therefore, atrium-specific LTCC expression, phosphorylation, and biophysical properties, and cardiomyocyte ultrastructural remodeling, should be addressed in future studies specifically designed and powered to assess sex as a biological variable. Finally, patient medications may have influenced ion channel expression or function and could not be fully controlled.

## Conclusion

In conclusion, this study demonstrates that downregulation of *I*_Ca,L_ in AF is governed by chamber-specific stress-induced degradation of membrane structures, leading to a reduced number of functional LTCCs that is not fully compensated by increased channel activity. Because atrial contractile dysfunction contributes to thrombus formation and stroke risk in AF,^8^ therapeutic strategies aimed at preserving or restoring atrial cytoarchitecture may improve *I*_Ca,L_, enhance atrial contractility, and reduce adverse clinical outcomes following cardioversion to SR. In addition, maintaining caveolae-associated signaling integrity may normalize LTCC activity and reduce atrial arrhythmogenesis.^45^ By integrating human patient data with computational modeling, our findings provide a mechanistic framework linking nanoscale membrane remodeling to emergent electrophysiological dysfunction in AF, supporting translational efforts to target microdomain-specific pathways in human disease.

## Disclosures

The authors have no conflicts of interest to disclose.

## Footnotes

Appendix Supplementary data associated with this article can be found in the online version at https://doi.org/10.1016/j.hrthm.2026.03.1953.
