# Supplementary material for: Downregulation of L-type Ca2+ current in patients with atrial fibrillation results from altered channel gating and disruption of membrane microdomains
Source: Heart Rhythm. 2026 Jul;23(7):e1170–82. doi: 10.1016/j.hrthm.2026.03.1953 (PMC13235752; doi:10.1016/j.hrthm.2026.03.1953)
Supplement: Supplementary Material [file mmc1.docx]

**SUPPLEMENTARY MATERIAL**

**Downregulation of L-type Ca^2+^ current in patients with atrial fibrillation results from altered channel gating and disruption of membrane microdomains**

**Authors:** Marina Balycheva, Benedict Reilly-O’Donnell, Anita Alvarez-Laviada, Kelly Zhang, Marta Mazzola, Jose L. Sanchez-Alonso, Carla Lucarelli, Roman Y. Medvedev, Cristina E. Molina, Sophie Schobesberger, Ivan Diakonov, Peter T. Wright, Claire E. Poulet, Nadja I. Bork, Natalia A. Trayanova, Giuseppe Faggian, Prakash Punjabi, Viacheslav O. Nikolaev, Alexey V. Glukhov, Julia Gorelik

**Supplementary Methods**

***Ethical approval***

All the IRB protocols were approved by the corresponding institutional review boards. Human atrial samples were obtained from patients undergoing coronary artery bypass surgery and/or mitral valve repair/replacement with or without concomitant atrial fibrillation ablation at Hammersmith Hospital, UK (Ethical approval number 12/WA/0196, REC Wales) and University of Verona, Italy (Prof.847CESC-Prot.13371 AOUI) and have therefore been performed in accordance with the ethical standards laid down in the 1964 Declaration of Helsinki and its later amendments. All persons gave their informed consent prior to their inclusion in the study. Normal human hearts that went unused for organ transplant were obtained from the University of Wisconsin Organ Procurement Organization, Madison, Wisconsin, USA, as approved by the University of Wisconsin institutional review board.

***Patient groups***

A total 112 patients with preserved left ventricular function undergoing open heart surgery were studied. There were 51 sinus rhythm (SR) male and female patients and 61 atrial fibrillation (AF) males and females. Concomitant heart diseases were coronary artery disease and mitral valve disease. The duration of AF was obtained by history taking and serial electrocardiograms. Only patients with persistent AF (longer than 6 months in anamnesis) were included in present study. Right atria (RA) and left atria (LA) chamber sizes and ejection fractions were recorded during echocardiography. Some patients were prescribed calcium channel and beta-adrenergic receptor blockers for the control of ventricular rate in AF. A subset of SR samples (n=8) utilized for structural and biochemical analysis were obtained from University of Wisconsin-Madison and approved by Institutional Review Board. Normal human hearts that went unused for organ transplant were obtained from the University of Wisconsin Organ Procurement Organization, Madison, WI, USA. At the time of harvest, hearts were aseptically excised, perfused and stored in cold cardioplegia solution, and transported on ice. Hearts were processed within 12 hours of explant.

***Human tissue samples and myocyte isolation***

Pieces of RA and/or LA appendages of 0.1 to 0.5 g were obtained during the coronary artery bypass surgery and/or mitral valve replacement procedure at Hammersmith Hospital, Imperial College London, UK, and School of Medicine, University of Verona, Italy. The ethics committees of Imperial College Healthcare NHS Trust and School of Medicine, University of Verona, Italy had approved experimental protocols. Patients gave written informed consent. Except for whole cell electrophysiology and Westrn blot experiments, LA samples were available only for AF group. After excision, atrial appendages were either used for myocyte isolation or were flash-frozen in liquid nitrogen for biochemical studies. The collected samples were cardioplegically arrested and cooled to 4-7^o^C in the operating room. The samples were maintained at 4-7^o^C to preserve tissue during ~10 minutes delivery from the operating theatre to the research laboratory. Atrial myocytes were isolated by enzymatic digestion as previously described (1). Briefly, individual specimens were transferred to ice-cold calcium free Krebs-Ringer saline solution consisting of (in g/L): 7.012 NaCl, 0.402 KCl, 1.332 MgSO_4_, 0.55 Pyruvate, 3.603 Glucose, 2.502 Taurine, 2.383 HEPES, 1.286 Nitrillotriacetic Acid; pH = 6.96. Connective and adipose tissue were removed and approximately 500 mg of myocardial tissue was minced with razor blades in small cubes (approx. 1-2 mm^3^). Then, the tissue pieces were washed with fresh Ca^2+^-free Krebs-Ringer solution 3 times for 3 min each at 37^o^C. After wash, cardiac tissue was incubated for 25 min in 10 ml of Krebs-Ringer solution containing (in g/L): NaCl 7.012, KCl 0.402, MgSO_4_ 1.332, Pyruvate 0.55, Glucose 3.603, Taurine 2.502, HEPES 2.383; pH = 7.4, supplemented with 200 nM CaCl_2_ and Proteinase type XXIV (0.36 mg/ml; Sigma-Aldrich) under gentle agitation. The partially digested tissue was transferred to 10 ml of Krebs-Ringer saline supplemented with collagenase type XIV (1 mg/ml Sigma-Aldrich). The tissue was incubated thrice with this solution for 10 min each at 37^o^C with gentle agitation. Usually, cardiomyocytes were visible by phase contrast light microscopy after the first incubation step, with the biggest amount of cells after the second incubation step. After each incubation step, the supernatants were transferred to a tube and centrifuged at 600 rpm for 3 min. The pellets were re-suspended in 2-3 mL of Krebs-Ringer solution. After isolation, human cardiomyocytes were plated following the same protocol as rat cardiomyocytes.

***Whole-cell electrophysiological recordings***

Macroscopic Ca^2+^ currents were recorded using the whole-cell patch-clamp configuration with the external recording solution of the following composition (in mmol/L): 120 Tetraethylammonium-chloride, 10 CsCl, 10 Glucose, 10 HEPES, 2 MgCl_2_, 4 CaCl_2_, pH 7.4 with CsOH. An internal pipette solution contained (in mmol/L): 100 Cs-methanesulfonate, 40 CsCl, 10 HEPES, 5 EGTA, 2 MgCl_2_, 5 Mg-ATP, pH 7.2 with CsOH. Patch pipettes had mean resistances of 3–4 MΩ. Currents were recorded using an Axopatch-1D amplifier connected to a Digidata1322A acquisition system (Axon Instruments, Foster City, CA, USA). The bath was connected to the ground via an Ag–AgCl pellet. Data were low-pass filtered at 2 kHz using the built-in Bessel filter of the amplifier and sampled at 10 kHz. All recordings were performed at room temperature (22-24°C). Depolarizing pulses from a holding potential of -40 mV to test potentials ranging from –45 mV to +60 mV with a duration of 150 ms were applied. Results were analyzed offline using pCLAMP10 (Axon Instruments) and OriginPro8.6 (OriginLab) software packages. Series resistance and whole cell capacitance were electronically compensated between 70 and 80% for each cell. Current amplitude at 10 mV was taken as a peak current for each cell. This value was divided by cells capacitance and was termed Ca^2+^ current density. Mean current values ± SEM were plotted as current-voltage (I-V) relationship. *I*–*V*’s were fitted with the modified Boltzmann equation, *I* = [*G*_max_× (*V*_m_−*E*_rev_)]/{1 + exp[(*V*_m_−*V*_0.5a_)/*K*_a_], where *V*_m_ is the test potential, *V*_0.5a_ is the half-activation potential, *E*_rev_ is the extrapolated reversal potential, *G*_max_ is the maximum slope conductance and *K*_a_ reflects the slope of the activation curve.

***Super-resolution scanning patch-clamp with pipette clipping modification***

After generating a topographical image of the cell surface by SICM, the tip diameter of the pipette is widened by clipping (2) to increase the area of attachment. Controlled widening of the scanning nano-pipette tip is described in details in (2). Briefly, the nanopipette tip-clipping procedure consisted of three steps. First, the pipette was navigated to a previously identiﬁed area of the coverslip free of cardiomyocytes. Second, the fall rate (the rate at which the pipette repeatedly approaches the surface during ‘‘hopping’’) was increased from the standby rate (typically 60 nm/ms) by approximately one order of magnitude (to ~500 nm/ms). At this fall rate, the noncontact mode of hopping probe could no longer be preserved because of the inherent latency of the z axis piezo feedback control. As a result, the pipette repeatedly crashed into the coverslip, clipping its tip and increasing its diameter because of the conical shape of the pipette. Pipette tip clipping resulted in stepwise increases of the pipette current as its resistance dropped (2). The clipping was automatically stopped by returning the fall rate to baseline (60 nm/ms) once the pipette current reached a desired level. This process could be repeated to ﬁne-tune the desired pipette tip diameter in steps as small as 10% by varying the stop criteria for current increase, duration and “clipping” fall rate.

Importantly the controlled clipping procedure did not change the overall shape of the pipette tip but reliably allowed the inner tip diameter to be increased approximately 4-fold: from 107 ± 16 nm to 417 ± 48 nm (2). The experimentally determined relationship between pipette resistance and inner pipette tip diameter for both intact and widened pipettes was in close agreement with theoretical predictions based on the tip geometry. On average the resistance of the widened pipettes was decreased ~2.4-fold (from 92.2 ± 8.9 MΩ to 38.7 ± 4.0 MΩ), thus making the modiﬁed pipettes more suitable for whole-cell patch-clamp recordings.

***Single channel electrophysiological recordings and data analysis***

Cell-attached patch-clamp recordings were performed at room temperature as previously described (3). Single-channel recordings were obtained using super-resolution scanning patch-clamp method with pipette clipping modification. After generation of a topographical image of the cell surface by SICM, the tip diameter of the pipette was widened from ~100 to ~350 nm. After clipping, the pipette was moved back and placed to a precise coordinate, centered at a site of known topology, T-tubule opening or crest of sarcolemma, and then sealed to the membrane for recording of functional LTCCs in cell-attached mode. Then non-contact mode of SICM was switched off and the pipette was lowered using the piezo actuator until it touched the cell membrane. Negative pressure was applied to get a giga-seal between the pipette and the membrane. All electrophysiological recordings were performed in the cell-attached patch-clamp configuration with resistance more than 4 GΩ and a current leak less than 2 pA. For recording of Ca^2+^ channels, cardiomyocytes were bathed in an external solution containing in (mmol/L): 120 K-gluconate, 25 KCl, 2 MgCl_2_, 1 CaCl_2_, 2 EGTA, 10 Glucose, 10 HEPES, pH 7.4 with NaOH, ~300 mOsm. Pipettes (borosilicate glass) were filled with an internal recording solution containing in (mmol/L): 90 BaCl_2_, 10 HEPES, 10 Sucrose, pH 7.4 with TEA-OH, ~280 mOsm. Axopatch 200A patch-clamp amplifier (Molecular Devices, USA) were used to record ion channel activity. Cell-attached currents were digitized using Digidata 1200B and acquired using a pClamp 10 data acquisition system (Axon Instruments; Molecular devices).

Single L-type Ca^2+^ channels (LTCCs) were identified and characterized by their voltage dependent properties. For this purpose depolarizing pulses from a holding potential of -80 mV were elicited to test potentials between -20 and + 20 mV. Analysis was performed as previously described (3). Single channels were sampled at 10 kHz and filtered at 2 kHz (- 3 dB, 8-pole Bessel). Single channel data were analysed using Clampfit version 10.2. Channel conductance was calculated by plotting the amplitude of fully resolved openings against the test potential for every single experiment. Applied voltage was corrected for a liquid junction potential of -16.7 mV.

Open probability (P_O_) was calculated from at least 10 consecutive sweeps. Peak current was calculated as the maximum of the overall average current from one single channel. In case of multiple-channel patches, n was derived from the maximum current amplitude divided by the number of channels in the patch.

Single channel current recordings were initially examined for presence of sub-conductance states using all points’ histograms as shown before (1,4,5). Most patches exhibited three to four conductance levels which were used for analysis and were named as levels 1 to 4. Each recording was analyzed separately for the conductance levels 1-4 using a threshold appropriate to the substate amplitude of openings. Openings that reached the 90% threshold for a given conductance state and events were also smaller than the next sub-conductance level were included. Single brief events shorter than 1ms were not included in the analysis.

A subset of LTCC recordings obtained from SR patients was reported previously (1). In the present study, these recordings were reused and reanalyzed to extract additional single-channel parameters that were not included in the prior publication. Specifically, LTCC open probability (P_O_) and related kinetic measures were newly quantified and used as control data for comparison with AF samples. No previously published analyses or summary statistics were duplicated, and all newly reported parameters represent original analyses performed for the purposes of the present study.

***T-tubule labeling***

T-tubule density was measured after sarcolemmal membrane labeling with Di-8-ANEPPS as described previously (6,7). Cardiomyocytes were incubated with 10 mM Di-8-ANEPPS (Molecular Probes, Eugene, OR, USA) for 1 min and then washed for 3 min before being observed under the confocal microscope. After Di-8-ANEPPS labeling, the density of T-tubules was quantified by the ratio of T-tubule fluorescence (T-tubule membrane) to total plasma membrane fluorescence (total membrane) in the same confocal slice, with excitation at 488 nm and emission detected at 520 nm. The T-tubule density was calculated by converting the Di-8-ANEPPS signal to a binary signal, using the autothreshold function of ImageJ. After exclusion of the surface sarcolemma, the whole z-series was analysed to provide the percentage stained. This was represented as T-tubule density.

***Western Blotting***

The tissue (around 30 mg) was cut in small pieces and the protein expression levels were evaluated in total lysate. Samples were homogenized in RIPA buffer (10 mM Tris pH 7.2, 150 mM NaCl, 0.1 % SDS, 1% Triton-X100, 5 mM EDTA, 100 μM Na3VO4, 10 mM NaF) containing protease inhibitor 1X (Thermo Fisher Scientific), using the TissueLyser II (Qiagen). Equal amounts of proteins were loaded into acrylamide gel, separated by electrophoresis, and transferred to a nitrocellulose membrane (Millipore). Immunoblotted signals were measured using antibodies against: caveolin-3 (BD 610421), pThr-286 CaMKII (1:500, MA1-047, Invitrogen), pSer-16 PLN (1:1000, A010-12, Badrilla). Data was normalized to GAPDH (Cell signaling 2118) used as a loading control. Densitometry analyses was performed using Image Lab software (Bio-Rad) and the signals were quantified using the Image J software.

***Cav3 overexpression protocol***

After isolation, human atrial cardiomyocytes were plated on laminin-coated dishes. 2 hrs after plating, cardiomyocytes were infected for 48 hrs with caveolin-3 (Cav3) adenovirus vector at a multiplicity of infection 300. Non- infected, but cultured (48 hrs), cardiomyocytes were used as a control in these experiments.

***Immunofluorescent labeling***

After 48 hrs culturing, cells were washed once with PBS and fixed with paraformaldehyde (4%) for 15 min at room temperature. Cells were permeabilized with Triton X-100 (0.5% in PBS) for 20 min at room temperature. After washing three times with PBS, cells were incubated in blocking buffer (PBS containing BSA, 1%; Glycine, 0.5 M; FCS, 4%; Tween 20, 0.1%) for 30 min at room temperature. Anti-Caveolin-3 (1:300 dilution, mouse, BD #610421) and anti-human Ca_V_1.2 (1:200 dilution, rabbit, Alomone #ACC-022) were used as primary antibodies (4°C, overnight). As secondary antibodies Alexa Fluor 633 goat anti-mouse (1:500 dilution, ThermoFisher #A21050) and Alexa Fluor 514 goat anti-rabbit (1:500 dilution, ThermoFisher #A31558) were used.

***Computational modelling of changes associated with AF***

For our computational studies, the human atrial action potential and Ca^2+^ traneinst model by Grandi *et al*. (8) was modified to reproduce the experimental LTCC behavior for LA and RA cardiomyocytes. The original ionic model includes the junctional cleft and a sarcolemmal compartment, with separate LTCC formulations (T-LTCC and C-LTCC) contributing to each subspace. These formulations, however, were dependent on the same set of steady-state activation and inactivation gating equations. Hence, to incorporate the microdomain-specific changes in channel function, two different sets of gating equations were created for the two channel subtypes. The current and gating equations were modified to fit the *I-V* curves produced by the model to our experimental data for RA and LA cells from patients in SR. First, the activation curves of both T-LTCCs and C-LTCCs were equally shifted toward positive clamp voltages to obtain the measured peak current densities at +15 mV. The gating equations’ slope factors, which characterize the steepness of voltage dependence of current activation, were then tuned according to the *I/I*_max_ vs. clamp potential data. Finally, the inactivation gating curves in the model were shifted so that the open probabilities of the T-LTCCs and C-LTCCs – calculated as the fraction of channels with activation and inactivation gates simultaneously open – followed the measured data (Table S1). These experimental recordings were taken at -6.7 mV to maintain a balance between distinguishable peak amplitudes and sufficient channel openings for our protocol. The values were then scaled to open probabilities at +15 mV based on Hullin *et al*. (9) so that the conductance from maximal channel openings could be adjusted to produce the experimental peak current densities (Table S2).

To incorporate the observed microdomain changes for cells from patients in AF, three new model parameters were added (Fig. S1). *TTdensity_scf* was used to fractionally scale the T-LTCC current, other currents contributing to the junction subspace, and the subspace volume itself according to experimental T-tubule density data. In AF, T-tubule density was decreased by 23% in the RA and 70% in the LA. Parameters *ICaL_junc_scf* and *ICaL_sl_scf* were used to modify the ratio of T-LTCC to C-LTCC current based on localized channel occurrence. For the RA in AF, there was a 26% decrease in tubule occurrence and 70% decrease in crest occurrence; for the LA in AF, there was a respective 76% and 69% decrease. The voltage sensitivities of the activation gates were further shifted so the maximum current densities of the *I-V* curves produced by the model occurred at the experimentally measured +24 and +20 mV for the RA and LA. Using the previously described approach, the inactivation gates were also shifted to reflect the significant increase in channel open probabilities. With these changes, the maximal conductances were again adjusted so that the peak current density matched our experimental data.

After parameterization, the model was used to help investigate the influence of different biophysical and cell ultrastructure parameters on the outcome of global calcium entry in AF myocytes.

***Statistical analysis***

Continuous variables were summarized as mean ± SEM for the given number of experiments, where N denoted the number of patient samples and n refers to the number of measurements; categorical variables were shown as frequency and percentage. Statistical analysis was carried out using an unpaired Student t-test or Mann – Whitney after test for normality (Shapiro-Wilks test). Categorical variables were compared with Fisher’s exact test. A value of *P*<0.05 was considered as significant. Where more than two unrelated conditions were evaluated, a One-way ANOVA with a Bonferroni’s post-hoc test was applied.

**References**

1. Glukhov AV, Balycheva M, Sanchez-Alonso JL et al. Direct Evidence for Microdomain-Specific Localization and Remodeling of Functional L-Type Calcium Channels in Rat and Human Atrial Myocytes. Circulation 2015;132:2372-84.

2. Novak P, Gorelik J, Vivekananda U et al. Nanoscale-targeted patch-clamp recordings of functional presynaptic ion channels. Neuron 2013;79:1067-77.

3. Bhargava A, Lin X, Novak P et al. Super-resolution Scanning Patch Clamp Reveals Clustering of Functional Ion Channels in Adult Ventricular Myocyte. Circ Res 2013;112:1112-20.

4. Cloues RK, Sather WA. Permeant ion binding affinity in subconductance states of an L-type Ca2+ channel expressed in Xenopus laevis oocytes. J Physiol 2000;524 Pt 1:19-36.

5. Gondo N, Ono K, Mannen K, Yatani A, Green SA, Arita M. Four conductance levels of cloned cardiac L-type Ca2+ channel alpha1 and alpha1/beta subunits. FEBS Lett 1998;423:86-92.

6. Lyon AR, MacLeod KT, Zhang Y et al. Loss of T-tubules and other changes to surface topography in ventricular myocytes from failing human and rat heart. Proc Natl Acad Sci U S A 2009;106:6854-9.

7. Sanchez-Alonso JL, Bhargava A, O'Hara T et al. Microdomain-Specific Modulation of L-Type Calcium Channels Leads to Triggered Ventricular Arrhythmia in Heart Failure. Circ Res 2016;119:944-55.

8. Grandi E, Pandit SV, Voigt N et al. Human atrial action potential and Ca2+ model: sinus rhythm and chronic atrial fibrillation. Circ Res 2011;109:1055-66.

9. Hullin R, Khan IF, Wirtz S et al. Cardiac L-type calcium channel beta-subunits expressed in human heart have differential effects on single channel characteristics. J Biol Chem 2003;278:21623-30.

**Table S1. Comparison of single-channel properties of tubular (T-LTCC) and extra-tubular (C-LTCC) L-type Ca^2+^ channels in RA and LA atrial myocytes from SR and AF patients.**

| Parameter | RA-SR | | RA-AF | | LA-AF | |
| --- | --- | --- | --- | --- | --- | --- |
| Subregion | T-LTCC | C-LTCC | T-LTCC | C-LTCC | T-LTCC | C-LTCC |
| Occurrence | 8/31  (29%) | 3/14  (21.4%) | 6/28  (21.4%) | **2/31**  **(6.45%) *** | **2/29**  **(6.9%) *** | 2/30  (6.7%) |
| Amplitude (pA) | 0.48±0.05 | 0.52±0.03 | 0.49±0.05 | 0.49±0.01 | 0.66±0.03 | 0.41±0.05 |
| Conductance (ps) | 13.7±3.0 | 13.7±0.7 | 14.0±2.2 | 10.9±0.3 | 9.95±0.01 | 6.2±0.9 |
| Availability (%) | 7.32±0.04  n=12 | 20.6±0.1  n=5 | **37.2±0.1 n=8 **** | **43.8±0.1**  **n=5 *** | 41.2±0.2  n=5 | 69.8±0.1  n=5 |
| P_open_ | 0.03±0.01 n=12 | **0.02±0.01 n=5 ^###^** | **0.15±0.02**  **n=8 ***** | **0.06±0.01 n=5 ^#,^***** | 0.14±0.04  n=5 | 0.16±0.05  n=5 |
| Mean open time (ms) | 0.89±0.05 n=20 | 0.63±0.04  n=6 | 0.99±0.05 n=16 | 0.67±0.09  n=8 | 0.81±0.07  n=5 | 0.94±0.08  n=4 |
| Mean closed time (ms) | 18.7±3.2 n=20 | 33.3±11.7 n=6 | **5.3±1.1 n=16 **** | **9.6±1.9**  **n=8 *** | 24.0±19.5 n=5 | 17.6±8.3 n=4 |

Occurrence shows a number of functional single channel activity which were found in all patch-clamp recordings (n/n). * – *P* < 0.05, ** – *P* < 0.005, *** – *P* < 0.001 vs. SR; ^#^ – *P* < 0.05, ^###^ – *P* < 0.001 vs. T-LTCC.

**Table S2: Open probabilities of single LTCCs scaled for computational modeling to different voltages from experimentally observed values taken at -6.7 mV.**

| Right Atrium | Experimental | Scaled to ~10mV | Scaled to ~20mV |
| --- | --- | --- | --- |
| P_O_ SR Junction | 0.03 | 0.06 | - |
| P_O_ SR Cleft | 0.02 | 0.04 | - |
| P_O_ AF Junction | 0.15 | - | 0.53 |
| P_O_ AF Cleft | 0.06 | - | 0.21 |

| Left Atrium | Experimental | Scaled to ~10mV | Scaled to ~20mV |
| --- | --- | --- | --- |
| P_O_ SR Junction | 0.03 | 0.06 | - |
| P_O_ SR Cleft | 0.02 | 0.04 | - |
| P_O_ AF Junction | 0.14 | - | 0.49 |
| P_O_ AF Cleft | 0.16 | - | 0.56 |


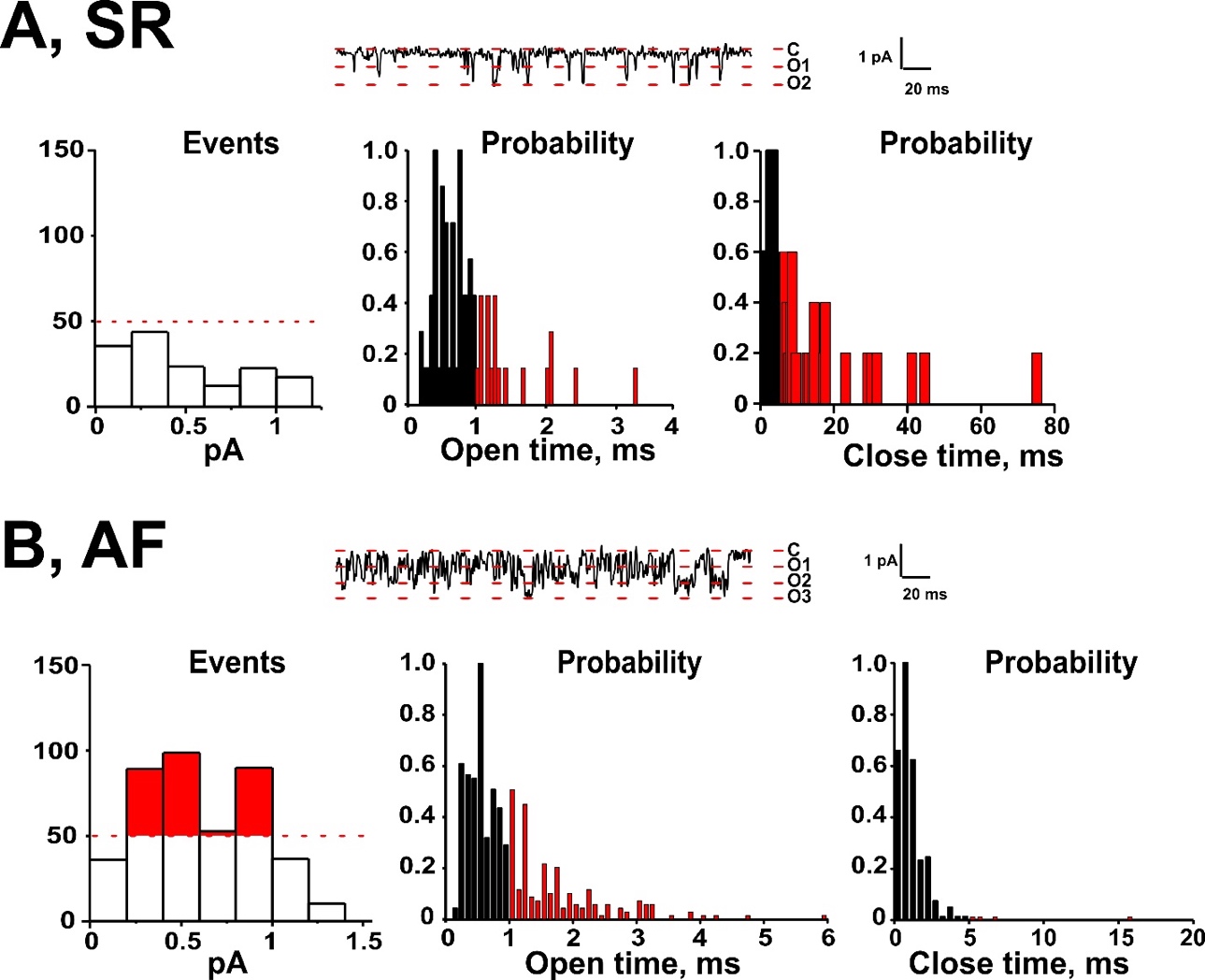


**Figure S1: Single T-LTCCs properties in RA myocytes obtained from SR and AF patients.** **A:** Representative traces at -6.7 mV showing multiple T-LTCC openings in SR. Lower panel shows number of events at different amplitude of openings and probability of open and close states, n=20. **B:** Representative traces at -6.7 mV showing multiple T-LTCC openings in AF. Lower panel shows number of events at different amplitude of openings and probability of open and close states, n=16.


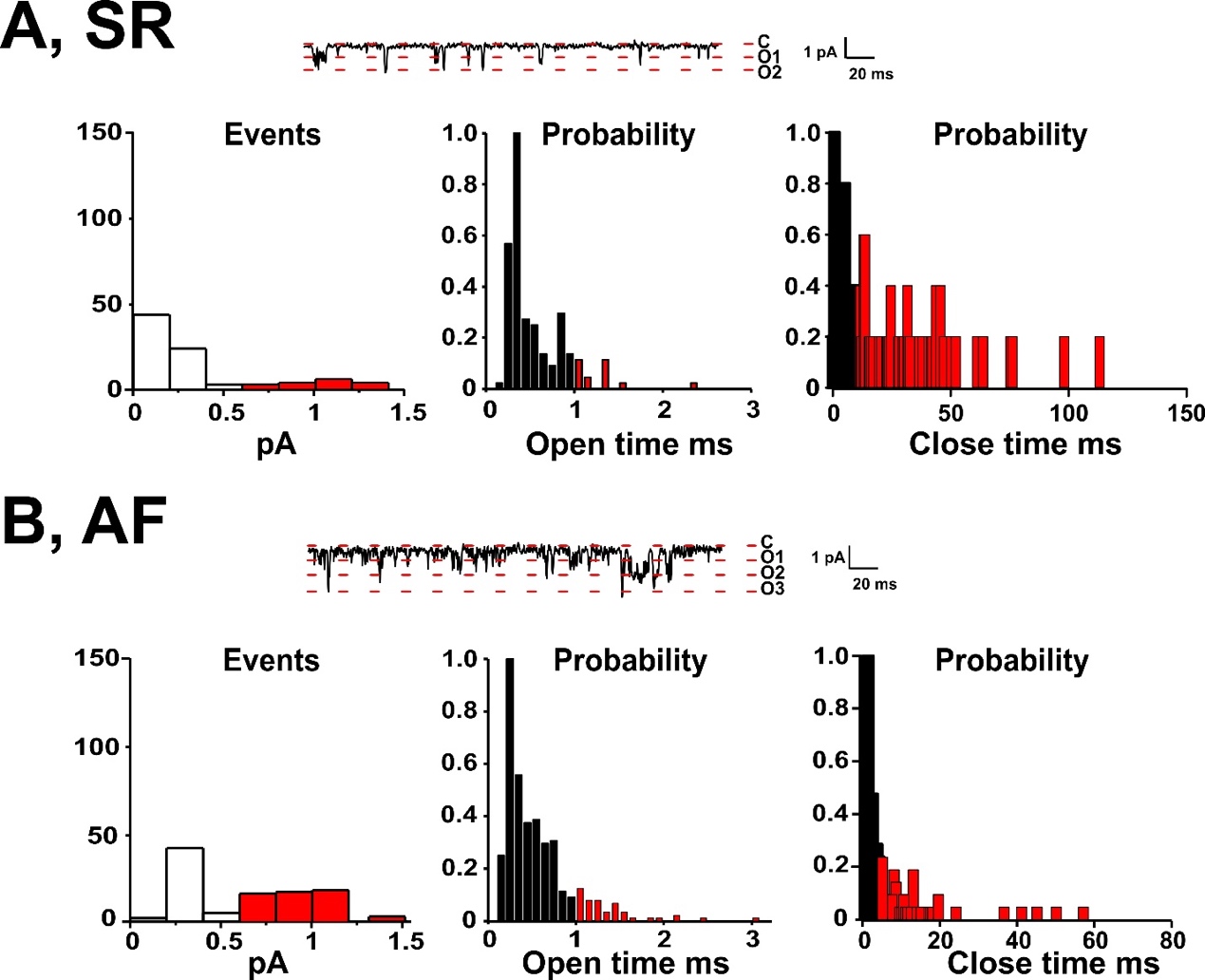


**Figure S2: Single C-LTCCs properties in RA myocytes obtained from SR and AF patients.** **A:** Representative traces at -6.7 mV showing multiple T-LTCC openings in SR. Lower panel shows number of events at different amplitude of openings and probability of open and close states, n=6. **B:** Representative traces at -6.7 mV showing multiple T-LTCC openings in AF. Lower panel shows number of events at different amplitude of openings and probability of open and close states, n=8.


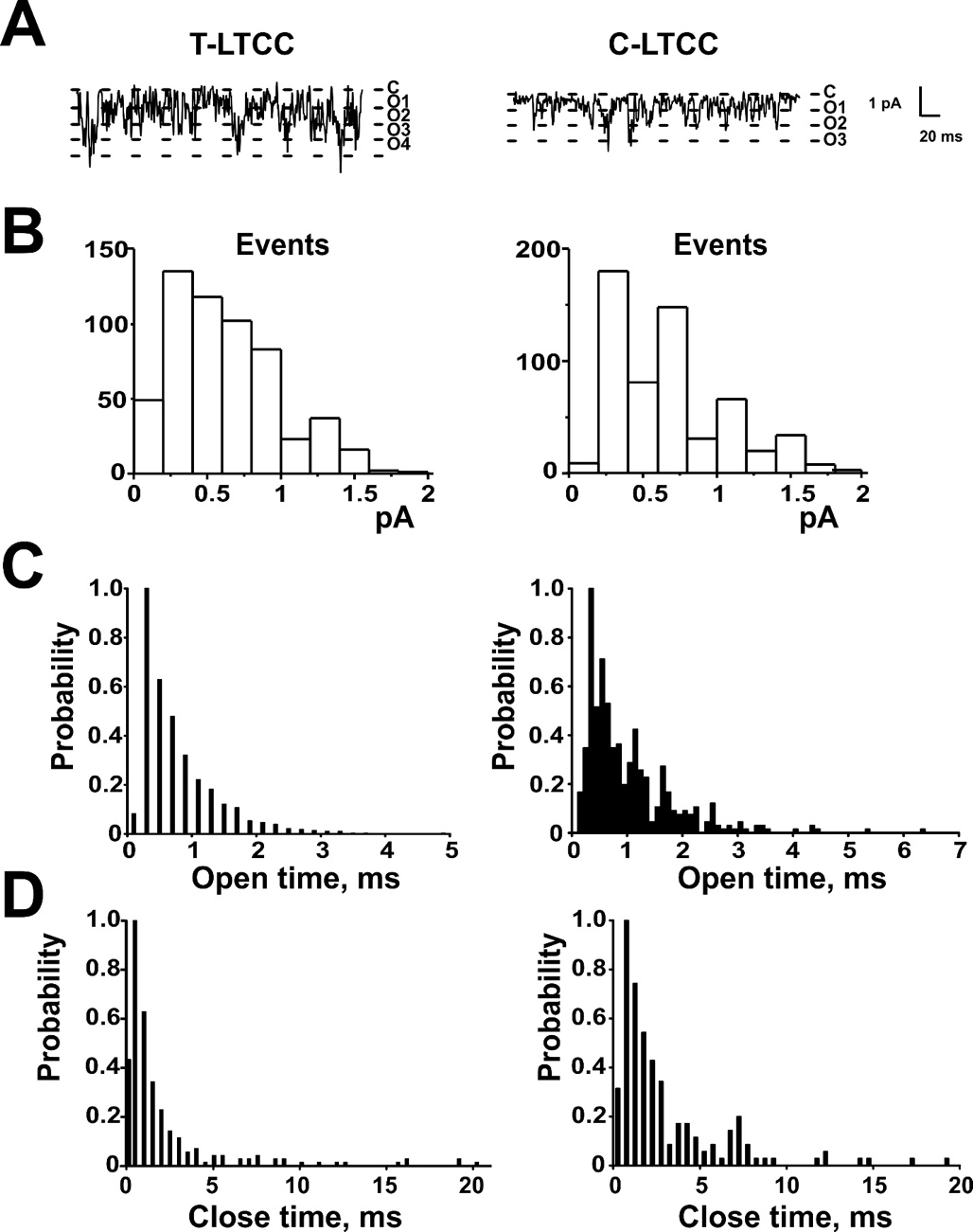


**Figure S3: Single T-LTCCs and C-LTCCs properties in LA myocytes obtained from AF patients.** **A:** Representative traces at -6.7 mV showing multiple T-LTCC openings in SR (*left*) and AF (*right*). **B through D:** Number of events at different amplitude of openings **(B)** and probability of open **(C)** and close **(D)** states are show for T-LTCC measured from SR (*left*) and AF (*right*) left atrial myocytes. N=5.
